# Seasonal Variation and Sources of Dissolved Nutrients in the Yellow River, China

**DOI:** 10.3390/ijerph120809603

**Published:** 2015-08-14

**Authors:** Yao Gong, Zhigang Yu, Qingzhen Yao, Hongtao Chen, Tiezhu Mi, Jiaqiang Tan

**Affiliations:** 1 Key Laboratory of Marine Chemistry Theory and Technology, Ocean University of China, Qingdao 266100, China; E-Mails: gongyaohh@163.com (Y.G.); zhigangyu@ouc.edu.cn (Z.Y.); chenht@ouc.edu.cn (H.C.); Tsing112@sina.com.cn (J.T.); 2 Tianjin Marine Environmental Monitoring Center, State Oceanic Administration People’s Republic of China, Tianjin 300450, China; 3 Key Laboratory of Marine Environment and Ecology, Ocean University of China, Qingdao 266100, China; E-Mail: mitiezhu@ouc.edu.cn

**Keywords:** Yellow River, nutrients, source, fluxes

## Abstract

The rapid growth of the economy in China has caused dramatic growth in the industrial and agricultural development in the Yellow River (YR) watershed. The hydrology of the YR has changed dramatically due to the climate changes and water management practices, which have resulted in a great variation in the fluxes of riverine nutrients carried by the YR. To study these changes dissolved nutrients in the YR were measured monthly at Lijin station in the downstream region of the YR from 2002 to 2004. This study provides detailed information on the nutrient status for the relevant studies in the lower YR and the Bohai Sea. The YR was enriched in nitrate (average 314 μmol·L^−1^) with a lower concentration of dissolved silicate (average 131 μmol·L^−1^) and relatively low dissolved phosphate (average 0.35 μmol·L^−1^). Nutrient concentrations exhibited substantial seasonal and yearly variations. The annual fluxes of dissolved inorganic nitrogen, phosphate, and silicate in 2004 were 5.3, 2.5, and 4.2 times those in 2002, respectively, primarily due to the increase in river discharge. The relative contributions of nutrient inputs to nitrogen in the YR were: wastewater > fertilizer > atmospheric deposition > soil; while to phosphorus were: wastewater > fertilizer > soil > atmospheric deposition. The ratios of N, P and Si suggest that the YR at Lijin is strongly P-limited with respect to potential phytoplankton growth.

## 1. Introduction

Rivers carrying suspended and dissolved materials from the land to the ocean are the principal link in transferring nutrients between these systems [1,2] and this greatly influences the aquatic ecology, especially in estuaries [3,4,5,6]. Globally, anthropogenic perturbations (e.g., wastewater and fertilizer inputs) have caused significant increases in fluvial nutrient fluxes [7], and have substantially modified coastal ecosystems [8,9,10]. In addition, the construction of riverine impoundments such as dams and reservoirs can modify the hydrology and consequently the fluvial transport of nutrients and sediments [11,12,13].

Previous studies have identified that the major sources of nitrogen and phosphate in the Yellow River (YR) were soil, fertilizer and wastewater inputs [14]. Owing to the substantial ongoing industrial and agricultural development in China, the nutrient fluxes in the YR have changed significantly over the last few decades. For example, the DIN fluxes varied from 0.92 × 10^9^ mol/a in the 1980s to about 7 × 10^9^ mol/a in 2002 [15]. Concentrations of dissolved inorganic nitrogen (DIN) and total phosphorus (TP) in the YR were higher than the world river background levels during the period from 1980–1989 [3,16]. Nutrient inputs via the YR also play an important role in the biogeochemical cycles in the coastal Bohai Sea. A substantial decrease in Si/N ratio in the Bohai Sea can be attributed to the rapid reduction of the YR discharge [15]. During 1950–2004, the maximum of discharge at Lijin was 973 × 10^8^ m^3^ and the minimum was 41.9 × 10^8^ m^3^. The decrease of discharge was mainly due to the climate change and human activities, such as reservoir construction and water utilization [17]. The main source of Si in the Bohai Sea was weathering and transport by the Yellow River. The fluxes of Si decreased with the decrease of the discharge, for the concentration of Si changed a little, but the concentration of DIN increased dramatically, due to the dramatic increase in fertilization [15]. Since 2002, the Yellow River Conservancy Commission has implemented a water-sediment regulation program at the beginning of every flood season to set up this scheme, in order to avoid a situation where there is no water flow and to improve the proportion between water and sediment transport by flushing the reservoirs and reducing sediment deposition in the lower reaches of the river. The dramatic changes in hydrology could also greatly influence the nutrient flux from the YR to the coastal Bohai Sea, especially after the implementation of large-scale water-sediment regulation schemes in recent years [18]. Although water quality monitoring for the YR commenced in the 1950s, systematic research on water quality was only undertaken in recent years [16,17,18,19,20], comprehensive nutrient data remain scarce, and further studies are necessary, especially following the implementation of formal water and sediment regulations in 2002 [21].

This paper reports the results of monthly monitoring at Lijin in the lower YR from the first year of water-sediment regulation in 2002 to 2004. The purposes of the investigation were to investigate the seasonal variations of the nutrients in YR, to identify and quantify the sources of nutrients to the YR, and to evaluate potential nutrient limitations in the YR.

## 2. Sampling and Methods

### 2.1. The Yellow River Basin

**Figure 1:**
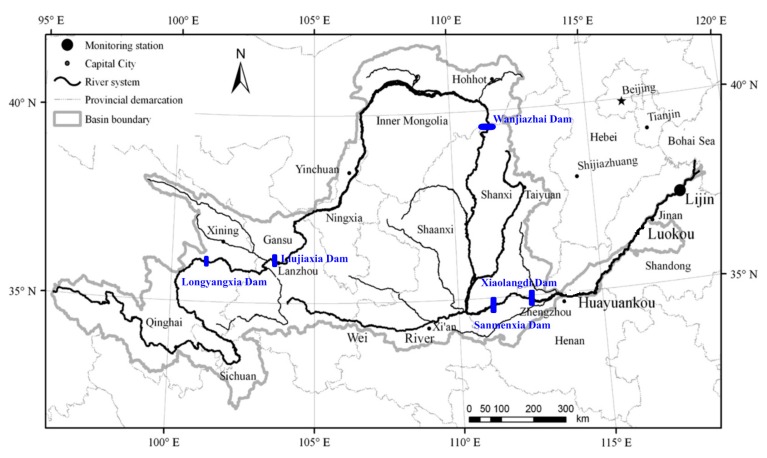
Location of the Yellow River Basin and sampling station.

The YR is, after the Yangtze River, the second largest river in China in terms of both length and basin area (Figure 1). The YR is approximately 5500 km long, originating in the northern part of the Bayankala Mountains of the Qinghai-Tibet Plateau at an altitude of 4830 m. Within the YR there are 168 large and mid-size reservoirs [22], which were located on the Yellow River and its tributaries in order to store water and regulate the river’s discharge and sediment. The YR drainage basin covers an area of 7.95 × 10^5^ km^2^, of which 1.19 × 10^5^ km^2^ or approximately 15% of total land area is farmland [23]. The farmland includes the arable lands, with wheat planted in the winter and the spring and corn planted in the summer and the autumn. The fertilizer used in the Yellow River basin had been listed in Table 1. The population of the YR basin is more than 107 M, which accounts for about 8.7% of the population of China [23]. The climate of the YR basin is arid, semi-arid to semi-humid with 80% of the annual precipitation falling in June–August each year [24]. The average basin-wide wet deposition was (basin-wide precipitation volume in parentheses) 404 mm (3.21 × 10^11^ m^3^) in 2002, 556 mm (4.4 × 10^11^ m^3^ ) in 2003, and 422 mm (3.4 × 10^11^ m^3^) in 2004, respectively [21].

The YR is known for its high sediment load, with an average value of 1.6 × 10^9^ tonne/a [25], approximately an order of magnitude greater than the Yangtze River [26]. The upper reaches of the A drain the northeastern part of the Qinghai-Tibet Plateau at an elevation between 3000–4000 m supplying ~60% of the river discharge but only ~10% of the sediment load. This area mainly comprises sandstone, dolomitic limestone and minor volcanics [27]. The loess plateau, which covers part of the upper reaches and most of the middle reaches of the YR comprises ~3 × 10^5^ km^2^, or ~40% of the total YR drainage basin [24]. The loess plateau, while contributing ~40% of the river discharge, with its easily erodible soils, contributes ~90% of the YR sediment load. A single rain event may typically constitute 10%–20% of the annual rainfall with short-term rainfall reaching up to 2 mm/min. With large intense precipitation, soil loss induced by individual storms may constitute 40% of the annual sediment load [24]. These features make the Chinese Loess Plateau the most physically eroded region of the world [28] with a physical erosion rate about 75 times greater than the chemical weathering rate [29]. The lower reaches of the YR traverse the fluvial plains of northern China 50–100 m above sea level. Typically, the river bed sits 5–10 m higher than the alluvial plain beyond the river banks due to the heavy riparian sedimentation [28].

**Table 1:** Fertilizer types used in provinces in the Yellow River drainage basin (×10^4^ t).

Province	Proportion (%) [14]	Nitrogenous Fertilizer [30,31,32]			Phosphorus Fertilizer [30,31,32]			Compound Fertilizer [30,31,32]		
		2002	2003	2004	2002	2003	2004	2002	2003	2004
Qinghai	22.8	3.3	3.13	3.0	1.6	1.55	1.6	2.0	1.85	1.8
Sichuan	3.5	118.5	117.47	120.2	42.3	41.87	42.9	37.8	37.46	39.3
Gansu	36.1	34.5	34.13	35.2	16.3	14.32	14.8	15.3	17.1	18.4
Ningxia	71.9	14.5	13.96	14.3	3.0	3.27	3.3	7.0	7.42	9.1
Neimenggu	10.7	45.9	50.45	54.2	14.5	15.61	18.3	17.9	21.43	24.9
Shanxi	62.2	40.8	39.79	40.5	19.3	18.62	18.9	23.2	24.96	26.9
Shaanxi	68.3	71.4	77.77	77.5	16.2	15.76	16.1	29.8	40.24	34.1
Henan	25.6	220.5	215.81	221.3	106.2	103.79	102.4	98.7	104.98	121.9
Shandong	15.1	189.7	182.03	185.3	54.6	54.49	57.7	149.4	154.82	164.1

### 2.2. Sampling and Methods

Water samples were collected monthly at Lijin in Shandong Province from 2002 to 2004 (except January 2003, Figure 1). The Lijin Hydrographic Station, located 100 km upstream from the YR estuary, is the last station before the river debouches into the Bohai Sea, and the records at Lijin represent the standard figures of the contributions of the YR to the sea. Therefore, the water samples collected at the Lijin Station can be used to examine nutrients concentrations delivered by the river to the sea.

Five equidistant surface sampling points were established in the main channel of the YR to obtain a representative sample. Because there is no tributary and sewage input into the YR below the Lijin Station, the nutrients fluxes at Lijin can represent the transport of YR to the Bohai Sea.

Water samples were collected directly into acid-washed 1 L high density polyethylene (HDPE) bottles, which were thoroughly rinsed with the sample prior to collection. The samples were filtered immediately through a 0.45-μm pore-size, pre-cleaned, cellulose acetate filter. Filtrates were stored in two 100-mL acid-cleaned low density polyethylene (LDPE) bottles and preserved with CHCl_3_. One sample for dissolved N and P analysis was kept frozen, and another for Si analysis, was stored in darkness at 4 °C.

Nutrient species in the filtrate were determined using an AA3 Continuous-Flow Analyzer (BRAN-LUEBBE, Hamburg, Germany). The quality of data was monitored by calibration with the national standards of China (GBW08623, GBW08632, GBW08637, GBW08640, GBW08648), and duplicate samples were determined for nutrients at μmol·L^−1^ level. The detection limits for phosphate (PO_4_^3−^-P), silicate (SiO_3_^2−^-Si), nitrite (NO_2_^−^-N), nitrate (NO_3_^−^-N), and ammonia (NH_4_^+^-N) were 0.024 μmol·L^−1^, 0.030 μmol·L^−1^, 0.003 μmol·L^−1^, 0.015 μmol·L^−1^, and 0.04 μmol·L^−1^, respectively. The daily discharge and sediment data were monitored by the YR Conservancy Commission [22].

### 2.3. Calculation of Nutrient Inputs from the Yellow River Basin

Major sources of nitrogen and phosphate in the YR include atmospheric deposition, soil loss from land, fertilizer loss and waste-water inputs [33]. Individual proportions of nitrogen and phosphorus inputs to the YR from the different sources were estimated as follows.

#### 2.3.1. Atmospheric Deposition

Atmospheric sources include both wet and dry deposition. There is little data about dry deposition, so we did not include it. Dissolved nitrogen and phosphorus from wet deposition were estimated using the equation:

*AD = r · W · A · C* (1)

where *AD* (g/a) is the atmospheric deposition of nutrients; *r* is the runoff coefficient, average 15%; *W* (m^3^) is the average basin-wide wet deposition with the values of (basin-wide precipitation volume in parentheses) 404 mm (3.21 × 10^11^ m^3^) in 2002, 556 mm (4.4 × 10^11^ m^3^ ) in 2003, and 422 mm (3.4 × 10^11^ m^3^) in 2004, respectively [22]; *A* (g/mol) is the atomic mass and *C* (g/m^3^) is the nutrient concentration in the wet deposition. The respective concentrations of nitrogen and phosphate in the wet deposition varied from 0.50 to 6.80 mg/L and from 3.41 to 14.9 μg/L, respectively [34].

#### 2.3.2. Input from Fertilizer Loss

Fertilizer loss from farmland is widely recognized as a major pathway for the increased flux of nitrogen and phosphorus into the YR [35,36]. Since the 1950s, agriculture has progressively developed within the YR basin with a corresponding increase in fertilizer application. The nitrogen fertilizer application was 420.80 × 10^4^ t, while it reached 789.70 × 10^4^ t in 1999. The fertilizer lost into the YR from farmland is the main form of non-point source pollution in the YR basin [37].

The amount of fertilizer loss within the YR basin to the river can be calculated according to the amount of fertilizer used (Table 1). Thus, we estimated the nitrogen and phosphorus loss from fertilizer within the YR basin from the total amount of fertilizer used and the fraction of nitrogen and phosphorus in each fertilizer; the net loss of fertilizer was calculated as:

(2) LF=l·∑p∑Mf

where *LF* (t) is the nutrient input to the YR from the fertilizer loss; *l* is the percentage loss of fertilizer within the YR basin, which were 15% for nitrogen and 2.0% for phosphate [38]; *p* is the fraction of the nutrients in the fertilizer, which is 0.352 for nitrogen fertilizer, 0.105 for phosphorus fertilizer [39], 0.15 for nitrogen in compound fertilizer, and 0.22 for phosphate in compound fertilizer [40]; *M* (t) is the amount of the fertilizer used in the nine provinces (Table 1) [30,31,32]; and *f* is the proportion of the land within the YR basin in the nine provinces [41].

#### 2.3.3. Nutrient Input from Soil Leaching

As outlined previously, the Loess Plateau exhibits substantial annual soil and water loss, accounting for approximately 90% of the total sediment in the YR [24]. This high suspended sediment load, via absorption mechanisms is likely to play an important role in the partitioning of total and dissolved nutrient concentrations. The amount of nutrient leached from soil is the product of the nutrient concentration and the sediment load:

*SL = S · C_NS_P_n_* (3)

where *SL* (t) is nutrient input to the YR from soil leaching, and *S* (t) is the sediment dischargedto the YR calculated from the annual suspended sediment load at Lijin. Using the suspended sediment load data from 1960 to 1985 of the middle reach of the YR (Luokou), which accounts for 90% of the basin, the relationship of suspended sediment load at Lijin with the sediment transport in the YR basin was *S = 2.262x + 1.551*, where *x* is the annual suspended sediment load (10^8^ t) at Lijin. The annual suspended sediment load measured at Lijin was 0.5 × 10^8^, 3.8 × 10^8^, and 2.7 × 10^8^ t in 2002, 2003, and 2004, respectively [22]. The background concentration of the nutrients in the soil (*C_NS_*) was estimated to be 0.8–1.5 g/kg for N, and 1.1–1.5 g/kg for P [26]. The fraction of DIN and DIP in the total soil (*Pn*), was 0.05 for nitrogen and 0.002 for phosphorus [42,43].

#### 2.3.4. Wastewater Nutrient Inputs

Wastewater includes both domestic sewage and industrial wastewater. The wastewater load is described in Section 4.1. The lowest concentrations of DIN and DIP from sewage treatment plants are 8.0 and 0.76 mg/L, respectively, while in the untreated wastewater, they could reach 60 and 9.0 mg/L [44]. The estimated nutrient load of wastewater is given by the relationship:

*WW = W · N* (4)

where *WW* (g) is the DIN or DIP load of wastewater; *W* (m^3^) is the wastewater transported to the YR, which were ×10^8^ m^3^ in 2002, 41.46 × 10^8^ m^3^ in 2003 and 42.65 × 10^8^ m^3^ in 2004, respectively [22]; and *N* (g/m^3^) is the concentration of DIN (8.0–60 mg/L) or DIP (0.76–9.0 mg/L) in the wastewater [44].

### 2.4. Nutrient Fluxes at Lijin

Nutrient fluxes in the YR at Lijin were estimated using the equation:

(5) $$F_{i}=\sum C_{ij}Q_{j}$$

where *F_i_* (g) is the annual flux of the species *i* in the river water; *C_ij_* (g/m^3^) is the average concentration of the species *i* in the river water during month *j*; and *Q_j_* is the cumulative discharge during month *j*.

## 3. Results

### 3.1. Discharge and Suspended Sediment

**Figure 2:**
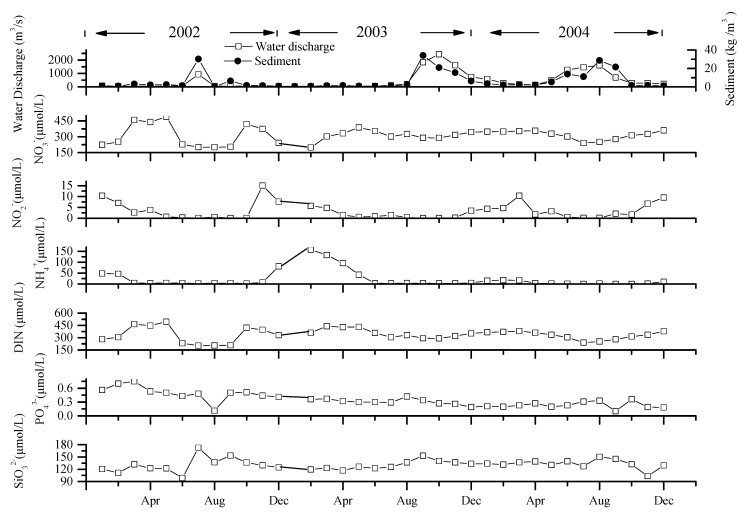
Water discharge, sediment and concentrations of nutrients at Lijin Station from 2002 to 2004.

Discharge in the YR in the period 2002–2004 varied substantially, with similar large variations of the suspended sediment load from 0.7 kg·m^−3^ to 34.2 kg·m^−3^. Discharge and suspended sediment load are strongly correlated (*R*^2^ = 0.69, *n* = 35), especially in summer (from June to August, Figure 2). Maximum discharge and suspended sediment load occurred in July 2002, October 2003, and August 2004, respectively. And the maximum discharge and suspended sediment load were more than 78 and 94 times of the minimum discharge and suspended sediment load, respectively. Summer discharge accounted for more than 70% of the total annual discharge. The annual discharge was 4.19 × 10^9^ m^3^/a, 1.926 × 10^10^ m^3^/a, and 1.988 × 10^10^ m^3^/a in 2002, 2003, and 2004, respectively [22], and the average value from 1956 to 2000 was 3.154 × 10^10^ m^3^/a [22].

### 3.2. Seasonal Variation of Nutrients

Seasonal and discharge-related variations of dissolved inorganic nitrogen (DIN) species (NO_3_^−^-N, NO_2_^−^-N, NH_4_^+^-N), dissolved inorganic phosphorus (DIP—PO_4_^3−^-P) and dissolved silicate (DSi—SiO_3_^2−^-Si) concentrations were significant in the YR at Lijin in the period of 2002–2004. Nitrate concentration varied from 201 to 491 μmol·L^−1^, with an average concentration of 311 μmol·L^−1^ in 2002, 313 μmol·L^−1^ in 2003, and 317 μmol·L^−1^ in 2004. Nitrite concentrations varied from 0.07 to 15.1 μmol·L^−1^, with an average concentration of 4.0 μmol·L^−1^ in 2002, 1.7 μmol·L^−1^ in 2003, and 3.8 μmol·L^−1^ in 2004. Ammonia concentrations varied from below the detection limit of 0.04 μmol·L^−1^ to 158 μmol·L^−1^ from 2002 to 2004, with average concentrations of 17.5 μmol·L^−1^ in 2002, 41.3 μmol·L^−1^ in 2003, and 5.9 μmol·L^−1^ in 2004.

**Figure 3:**
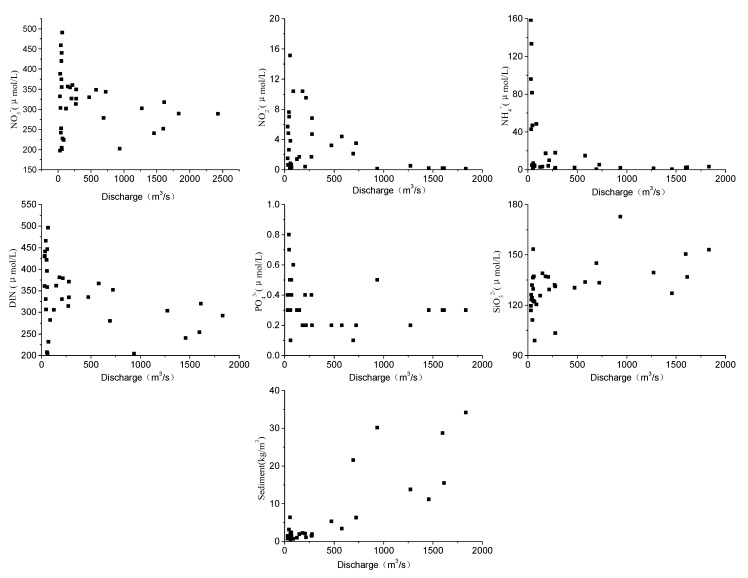
The relationship between nutrients, suspended sediment and discharge in the Yellow River.

Concentrations of both NO_2_^−^-N and NH_4_^+^-N were generally low during high discharge, but high during low discharge, thus exhibiting strong seasonal variations. The maximum concentration of NH_4_^+^-N was nearly 800 times of the minimum. The trend of NO_3_^−^-N, however, was different from the other forms of dissolved N species, displaying much less seasonality (Figure 2) and the concentration was typically between 200 and 350 μmol·L^−1^ at moderate to high discharge (Figure 3). NH_4_^+^-N, NO_2_^−^-N and NO_3_^−^-N attained the maximum concentrations successively in February, March, and April 2004. Nitrate was the major form of DIN (= the sum of NH_4_^+^-N, NO_2_^−^-N and NO_3_^−^-N), accounting for 73%–99%, 55%–99% and 93%–99% in 2002, 2003 and 2004, respectively.

Dissolved inorganic phosphorus (DIP) concentration varied from 0.10 to 0.75 μmol·L^−1^, with a mean value of 0.49 μmol·L^−1^ in 2002, 0.31 μmol·L^−1^ in 2003 and 0.23 μmol·L^−1^ in 2004, reflecting a substantial decrease during the study period. In most cases, DIP was within the range of 0.10–0.40 μmol·L^−1^ (Figure 2). The highest concentration of DIP occurred in March 2002 (0.75 μmol·L^−1^), and DIP was relatively low during high discharge period (Figure 3).

Dissolved silicate (DSi) concentration varied from 99 to 173 μmol·L^−1^, with an average value of 130 μmol·L^−1^ in both 2002 and 2003 and 133 μmol·L^−1^ in 2004. Maximum concentration of DSi occurred in July 2002, September 2003 and August 2004, while the minimum concentration occurred in June 2002, April 2003 and November 2004, respectively (Figure 2). In general, DSi displayed a positive relationship with both discharge and suspended sediment concentration (Figure 3).

As reflected by the significant, often flow-related variations of the nutrient concentrations, the molar DIN to DIP ratio in the YR water varied from 503 to 2804, with discharge-weighted averages (range in parentheses) of 576 (503–1861), 1132 (787–1848), and 1334 (769–2804) in 2002, 2003, and 2004, respectively. The increase in average molar DIN to DIP ratio from 2002 to 2004 reflects the substantial decrease of the DIP concentration during 2002 to 2004. The molar ratio of DSi to DIN ranged from 0.24 to 0.85, and, in contrast to average molar DIN to DIP ratios, similar averages of 0.67, 0.46, and 0.48 in 2002, 2003, and 2004, respectively due to the small relative changes in the concentrations of DSi and DIN.

## 4. Discussion

### 4.1. Factors Influencing Nutrient Concentrations

Major sources of nitrogen and phosphate in the YR include atmospheric deposition, soil loss from land, fertilizer loss and waste-water inputs [30]. Individual proportions of nitrogen and phosphorus inputs to the YR from the different sources were estimated in Table 2. Estimates of the relative proportions of DIN and DIPs for the period 2002 to 2004 were (Table 3): for DIN: wastewater > fertilizer loss > atmospheric deposition > soil loss from land, and for DIP were: waste-water > fertilizer loss > soil loss from land > atmospheric deposition. These results suggest that the primary factors influencing both DIN and DIP in the YR from 2002 to 2004 were wastewater discharge and fertilizer loss.

**Table 2:** Sources of nutrient inputs to the Yellow River from 2002 to 2004 (10^4^ t).

Source	DIN			DIP		
	2002	2003	2004	2002	2003	2004
Atmospheric deposition	2.44–32.9	3.34–45.1	2.54–34.3	0.017–0.072	0.024–0.103	0.016–0.70
Fertilizer	3.28–24.6	3.36–25.2	3.44–25.8	0.11–2.57	0.12–2.79	0.13–2.88
Soil leaching	1.07–2.01	4.06–7.61	3.06–5.74	0.059–0.08	0.22–0.30	0.17–0.23
Waste water	3.30–24.8	3.31–24.9	3.41–25.6	0.35–3.72	0.35–3.73	0.36–3.84

The environmental changes in the YR basin are heavily impacted by the population of over 100 million. From the 1980 to the present, the population in the YR basin has continuously increased [45]. Many studies have shown that, major factors contributing to nitrogen input in the basin include industry, agriculture (fertilizer) and population [46,47]. Over 100 million people live in the YR basin, most areas are agriculture-dominated. Therefore, the nitrogen transportation is mainly impacted by the population growth and fertilizer use, but less influenced by industrial discharges. In the late 1990s, China became the world’s largest producer, consumer, and importer of chemical fertilizers. The supply of chemical fertilizers from domestic sources has increased by four percent annually since 1980. Although China only contains 10 percent of the world’s arable land, it has consumed 25 percent of the annual global supply of chemical fertilizers since 2002. The average national per hectare application of chemical fertilizers in China reached 280 kilograms in 2000—three times the world average [48]. The YR drainage basin covers an area of 7.95 × 10^5^ km^2^, in which the area of farmland is 1.19 × 10^5^ km^2^, approximately 15% of total land area [49]. The use of chemical nitrogen fertilizer had sharply increased from 3.02 × 10^6^ ton in 1981 to 8.40 × 10^6^ ton in 2012. A recent report [50] showed that the quantity of wastewater from the YR drainage basin increased dramatically from ~2.0 Gt/a during the 1980s to 4.36 Gt/a in 2010. The DIN load in the YR system was largely attributed to the increasing use of chemical fertilizer and wastewater in the drainage basin.

**Table 3:** Nutrient fluxes in the Yellow River at Lijin Station in 2002, 2003 and 2004.

Year	Nutrient	NO_2_^−^-N	NO_3_^−^-N	NH_4_^+^-N	DIN	PO_4_^3−^-P	SiO_3_^2−^-Si
2002	Flux (mol·a^−1^)	7.75 × 10^6^	1.04 × 10^9^	3.62 × 10^7^	1.09 × 10^9^	2.03 × 10^6^	6.47 × 10^8^
	Flux (t·a^−1^)	1.08 × 10^2^	1.46 × 10^4^	5.07 × 10^2^	1.52 × 10^4^	0.63 × 10^2^	1.81 × 10^4^
2003 ^a^	Flux (mol·a^−1^)	1.07 × 10^7^	5.71 × 10^9^	1.08 × 10^8^	5.83 × 10^9^	5.34 × 10^6^	2.66 × 10^9^
	Flux (t·a^−1^)	1.50 × 10^2^	7.99 × 10^4^	1.52 × 10^3^	8.16 × 10^4^	1.66 × 10^2^	7.45 × 10^4^
2004	Flux (mol·a^−1^)	3.84 × 10^7^	5.69 × 10^9^	6.95 × 10^7^	5.80 × 10^9^	5.00 × 10^6^	2.69 × 10^9^
	Flux (t·a^−1^)	5.37 × 10^2^	7.97 × 10^4^	9.73 × 10^2^	8.12 × 10^4^	1.55 × 10^2^	7.54 × 10^4^

^a^ As there were no data for January 2003, the concentration of nutrients in February 2003 was used as a proxy for the concentration in January 2003.

The absorption of DIP onto the suspended sediments in the YR may be the major factor resulting in the relatively low concentration compared to other world rivers [51]. The concentration of DIP in the YR was close to mean value of the unpolluted rivers in the world [3] and less than one tenth that of polluted rivers in Europe and North America and also the Changjiang and Zhujiang Rivers [10,19,52,53]. Particulate phosphorus (PP) is the predominant species of total phosphorous (TP), which represents 89.2%–97.6% of TP in 2001 in the YR. Thus, when the suspended sediment load is high in the summer, the DIP concentration should be low due to the combined effects of dilution and absorption onto particle surfaces. In contrast, however, the DIP concentration in the summers of 2003 and 2004 were actually higher. Agriculture are well developed in the YR basin [23,24]. The middle and lower part of the YR basin are dominated by agriculture land-use. Nitrogen- and phosphorus-based fertilizers are used extensively in the YR basin, accounting for approximately 9% of the national consumption. The fertilizers are mainly used during the period of March to May and September to October. High DIP concentration was found during these periods (Figure 2). Thus, use of fertilizer may contribute to the seasonal variation of DIP. In addition, rainfall mainly occurred in summer in the YR basin, especially in 2003 and 2004 [22]. Furthermore, the increased wastewater discharge and fertilizer loss contributing inherently high DIP concentration surpassed the effect of dilution due to increased flow.

Silica is delivered to surface waters mainly by rock weathering, which releases dissolved SiO_2_ into drainage water with its abundance depending on both the lithological nature of the watershed and on the temperature [54,55]. Neal *et al.* [56] have pointed out that SiO_2_ concentration in headwaters could be in equilibrium with quartz/chalcedony levels. One of the unique geological features of the YR basin is the wide loess, which covers about 44% of the basin. The loess in the vast YR basin has a similar grain-size distribution, being dominated by 50%–60% silt (0.01–0.05 mm) and 20%–30% clay (<0.005 mm), with very few grains greater than 0.1 mm. Mineralogically, the loess is dominated by quartz, alkali feldspar, micas, and carbonate [57]. Waters running through loess and loess-like deposits can become enriched in SiO_2_.

### 4.2. Nutrient Fluxes at Lijin

**Figure 4:**
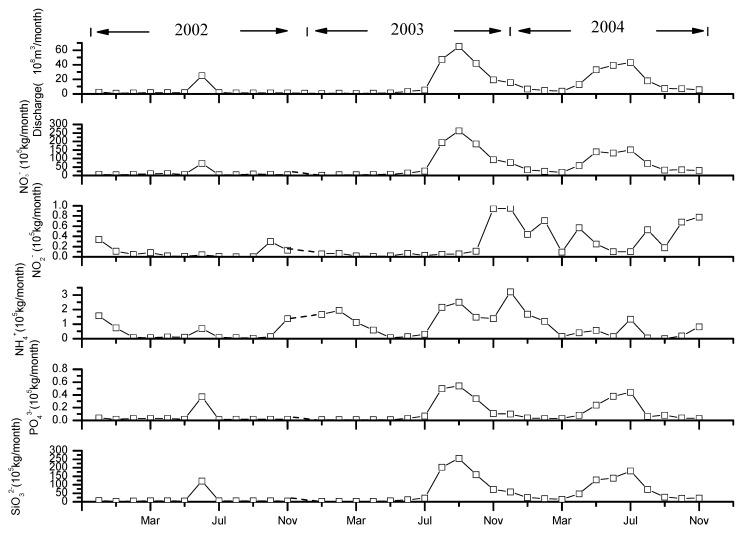
Monthly nutrient fluxes in the Yellow River from 2002 to 2004.

**Figure 5:**
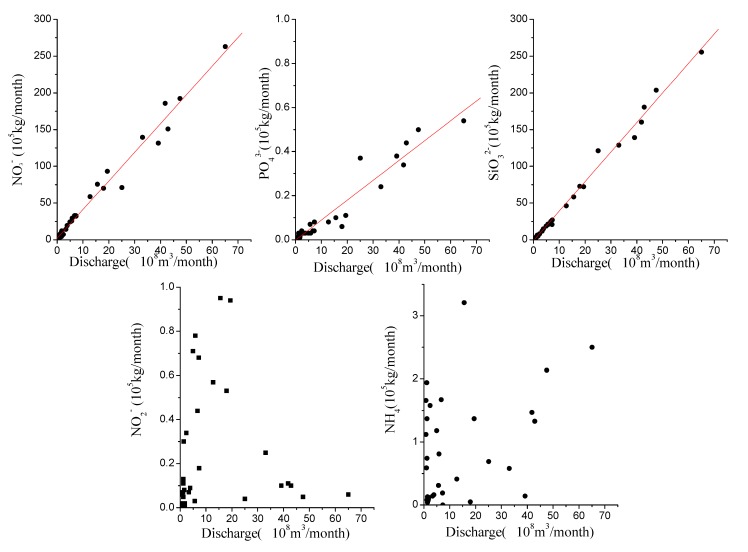
Relationship between nutrient fluxes and discharge from 2002 to 2004.

Estimated nutrient fluxes for 2002, 2003, and 2004 are shown in Table 3 and Figure 4 with large annual variations. In general, in 2003 and 2004 individual nutrient fluxes were substantially larger than in 2002. The flux of DIN in 2004 was 5.4 times that of 2002 with NO_3_^−^-N constituting the majority (~95%) of DIN. Increases in the fluxes of NO_3_^−^-N and SiO_3_^2−^-Si paralleled discharge, which in 2004 was 4.7 times that of 2002. Fluxes of DSi, DIP and nitrate were strongly correlated with discharge, in particular for DSi, DIP and NO_3_^−^-N and to a lesser extent NH_4_^+^-N, generally increased with flow, while the flux of NO_2_^−^-N, albeit highly variable at low flow, generally decreased with increasing flow (Figure 5, *R*^2^ = 0.98 for DSi, *R*^2^ = 0.92 for DIP, *R*^2^ = 0.98 for nitrate).

### 4.3. Nutrient Limitation and Seasonal Variability in the Yellow River

**Figure 6:**
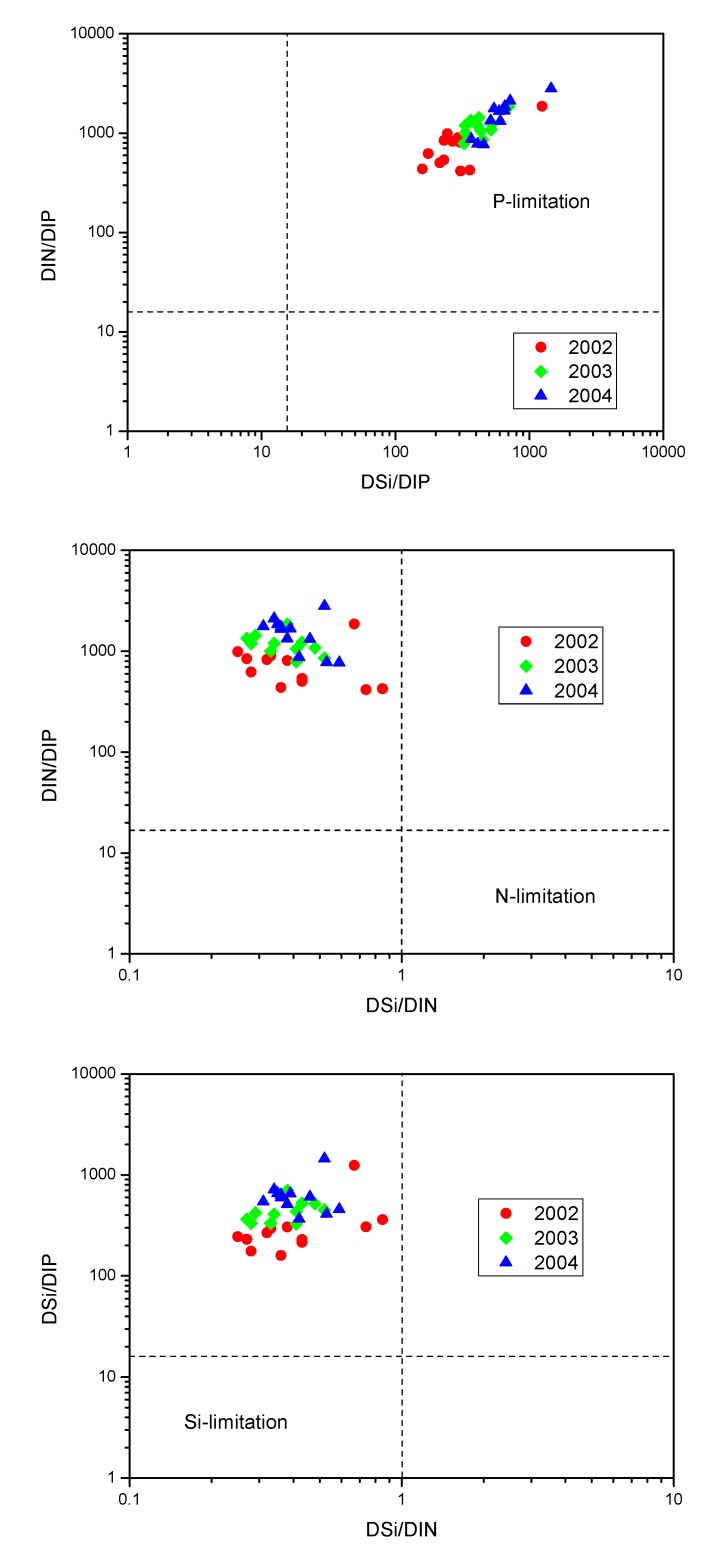
Nutrient limitation diagrams for 2002 to 2004 for the YR. Zones of potential P-, N- or Si limitation are marked. Dotted lines define Redfield ratios (Si:N:P of 16:16:1) of potential nutrient limitation [60].

Nutrient limitation diagrams using molar nutrient ratios derived from nutrient measurements from 2002 to 2004 in the YR are shown Figure 6 [58,59]. Zones indicative of potential nutrient limitation to phytoplankton growth based on the Redfield ratio are also shown [60]. For the years 2002 to 2004, there is evidence of strong potential P-limitation with no concurrent potential N- or Si-limitation, with respect to phytoplankton growth in the YR at Lijin. The observed P-limitation reflects the relatively high concentrations of both DIN and Si present in the river water. Dissolved concentrations of the three nutrients (expressed as DIP, DIN and DSi) are sufficiently high; however, in the absence of other limiting factors such as light, micronutrients or low temperatures, substantial phytoplankton biomass could eventuate. In addition, both high N and Si concentrations relative to P suggest that where phytoplankton biomass occurred, diatoms potentially predominated. The occurrence of P-limitation has also been noted in studies of nutrient concentrations and net fluxes in other Chinese rivers and coastal ecosystems [61,62] with the potential for significant modification to primary production [63].

**Figure 7:**
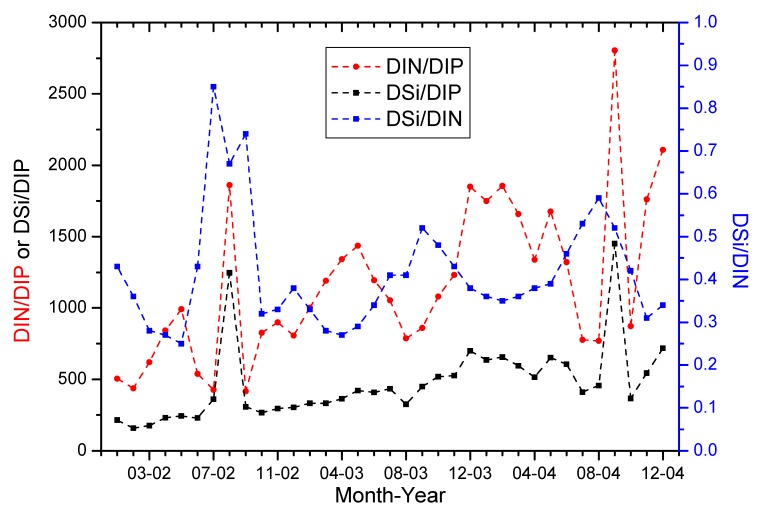
Temporal trends of dissolved nutrient ratios in the lower YR at Lijin, 2002 to 2004.

Temporal plots in molar nutrient ratios reveal features not readily observed in plots of dissolved nutrient concentrations alone (Figure 7). Both DIN/DIP and DSi/DIN exhibit a strong discharge-related periodicity although over the majority of the period 2002–2004 these trends are inversed. The DIN/DIP generally peaks in about March to April whereas the DSi/DIN generally peaks in about July and August, or about 6 months apart in low discharge and high discharge periods respectively. Temporal trends for DSi/DIP are generally more attenuate, however, there is a more general correspondence with increasing discharge and DIN/DIP. The DIN/DIP and DSi/DIN peaks were shown in August or September (in 2002 and 2004), when the discharge was high. It was the result of the diffuse, point sources and weathering processes. The diffuse process was the most important factor for DIP, while the weathering processes for DSi.

In addition to the marked periodicity observed for both DIN/DIP and DSi/DIN, there is also an increasing underlying baseline trend for all of the nutrient ratios that is most readily observable for DSi/DIP (Figure 7). Excluding the two large peaks on August 2002 and September 2004, the average annual increase in the underlying baseline ratio for DSi/DIP is approximately 100%, or a doubling per year. Increases in the baseline trends for DIN/DIP and DSi/DIN are more difficult to discern, however a similar increase is apparent for DIN/DIP whilst the DSi/DIN molar ratio increases by about 30% per annum. These changes in molar nutrient ratios correspond to a decline in DIP of approximately 30% per annum and potentially small decreases in DSi and DIN (Figure 2). If a strong decline in DIP concentrations is maintained subsequent to 2004, it is likely that potential P-limitation to phytoplankton growth within the lower YR will increase substantially. This trend is already apparent with increasing potential P-limitation from 2002 to 2004 (Figure 7).

Increases in DSi concentration closely correspond to increased sediment discharge, which occurred during 2003 and 2004 and would reflect this increasing predominance of soil-derived silica. DSi mainly comes from diffuse sources. Decreasing DIP reflected a decrease in the supply or increased retention upstream of Lijin. Low flow velocities, high transparency and high nutrient levels stimulate planktonic growth within reservoirs. Increased phytoplankton production and subsequent algal sedimentation are the key processes involved in the transfer of dissolved reactive phosphorus to particulate phosphorus in several dammed rivers [64]. Thus, phosphorus transformation and retention in the large reservoir might result in a reduction of phosphorus in the Yellow River. In contrast to the nitrogen and silicon contents, the DIP content appears to be low in the Yellow River. Changes in the DIN concentrations vary inversely with flow reflecting dilution from the source with the majority of DIN present as nitrate.

### 4.4. Comparison of the Nutrient Status of the Yellow River with Other Major World Rivers

Average dissolved nutrient concentrations in the YR differ substantially from other major world rivers (Table 4). The average concentration of nitrate in the YR from 2002 to 2004 of 314 μmol·L^−1^ was 40 times the world average for unpolluted rivers [3], substantially higher than polluted rivers in Europe and North America (Rhone River, Mississippi River, Lorie River *et al.*, Table 4) and other rivers within China, including the Changjiang and Zhujiang Rivers [19,52].

In contrast to nitrate, the major constituent of DIN within the YR, the concentration of DIP in the YR was close to the world average for unpolluted rivers [3] and less than one tenth that of polluted rivers in Europe and North America [10,52]. Compared with other Chinese rivers, the concentration of DIP in the YR was lower than in the Changjiang and Zhujiang Rivers [19,53], but higher than in the Yalujiang River [19]. The absorption of DIP onto the high sediment load of the YR may be a major factor influencing the lower DIP concentrations relative to DIN.

Average DSi concentration of 140 μmol/L in the YR was similar to the Loire, Seine, and Po Rivers, among other world rivers [12,52,65], but within China, higher than the Changjiang river [53], but lower than in the Yalujiang and Zhujiang Rivers [19].

**Table 4:** Comparison of nutrient concentrations in the YR with other major world rivers (μmol/L).

River	NO_3_^−^	PO_4_^3−^	SiO_3_^2−^	N/P	Reference
Amazon	10	0.7	115	14	[66]
Mississippi River	114	7.7	127	15	[10]
Loire	184	2.55	163	72	[52]
Rhone River	74.5	4.2	81.2	17.7	[67]
Seine	429	32.3	183	13	[12]
Po	150	4.6	120	32	[65]
Morlaix	397	3.90	138	101	[2]
Ob	56	2.3	164	24	[68]
Yenisey	26	0.4	107	65	[68]
Yukon	2.43	0.05	82	69	[69]
Changjiang	70.3	0.83	102	84	[53]
Zhujiang	62	0.75	150	46	[18]
Yalujiang	309.8	0.04	168.4	7745	[18]
YR	291	0.29	140	1003	This study
World river average	7.14	0.32	—	10.3	[3]

The N:P molar ratio in the YR of DIN and DIP was much higher than in other world rivers, being approximately one hundred times that of the world river average and more than ten times that of polluted rivers in Europe and America. This higher N:P molar ratio is principally due to the substantially higher dissolved nitrate concentration given the similar average dissolved phosphate concentrations in the YR and world rivers. Molar N:P ratios fertilizer used in China may typically range from 22–44 or even higher [53], while the estimated N:P molar ratio of input to the YR from fertilizer was 47.8, 45.0, and 44.6 in 2002, 2003, and 2004, respectively (Table 3). These high estimated N:P molar ratios may reflect the preferential leaching of nitrogen relative to phosphorus observed to occur the YR basin [70] in addition to substantial DIP uptake by suspended sediment.

## 5. Conclusions

From 2002 to 2004, the concentration of DIN (as nitrate, nitrite, and ammonia) in the YR showed a strong seasonal and flow-related variation. In contrast, concentrations of DIP and DSi were less sensitive to discharge. Notably, a strong decline in DIP concentration from 2002 to 2004 is apparent. Compared to other major world rivers, the YR was relatively enriched in NO_3_^−^-N but relatively poor in DIP. Concentrations of DSi were similar to major world rivers. In summary, the fact that nitrogen and phosphorus is mainly influenced by fertilizer loss and wastewater, provides important evidence on control options for pollution control both for the lower YR and for nutrient input to the YR estuary and to the Bohai Sea. We should transform the agriculture into an agriculture using less fertilizer and build more powerful wastewater treatment plants. The YR is enriched with DIN and very poor in phosphate. The molar N:P ratios of riverine nutrient has very high DIN:DIP. Nutrient transport from the YR can result in phosphorus limitation for phytoplankton growth in the YR estuary and the adjacent Bohai Sea.
